# CXCL1: a novel therapeutic target to increase aneurysm healing after coil embolization

**DOI:** 10.3389/fstro.2026.1746652

**Published:** 2026-02-10

**Authors:** Devan Patel, Melanie Martinez, Supreeya A. Saengchote, Kartik Motwani, William S. Dodd, Zahra Hasanpour Segherlou, Haiyan Xu, Koji Hosaka, Brian L. Hoh

**Affiliations:** 1 Department of Neurosurgery, University of Florida, Gainesville, FL, United States; 2 Department of Neurosurgery, University at Buffalo Jacobs School of Medicine and Biomedical Sciences, Buffalo, NY, United States

**Keywords:** aneurysm healing, coiling, CXCL1, intracranial aneurysm, macrophages, neutrophils

## Abstract

**Objective:**

CXCL1 is highly expressed in human aneurysms but its role in aneurysm healing is unknown. The objective of this study was to determine whether CXCL1 neutralization increases murine aneurysm healing post-coiling.

**Methods:**

Carotid artery aneurysms were created in female and male C57BL/6 mice. CXCL1 expression was compared between aneurysms and sham-operated carotid arteries. In a separate cohort, aneurysms were coiled with poly (lactic-co-glycolic acid) (PLGA)-coated coils. Mice received intraperitoneal injections of either CXCL1 neutralizing antibody or IgG control for 7, 14, or 21 days post-coiling. Coiled aneurysms were assessed for aneurysm healing, neutrophil infiltration, macrophage polarization, and total macrophage burden.

**Results:**

CXCL1 is highly expressed in murine carotid artery aneurysms. CXCL1 neutralization significantly increased aneurysm healing compared to IgG when administered for 14 days (*females:* 66.4% vs. 51.2%, *p* = 0.03; *males:* 69.8% vs. 47.0%, *p* = 0.004) and 21 days (*females:* 71.9% vs. 44.3%, *p* = 0.002; *males:* 67.8% vs. 61.6%, *p* = 0.02), but not when given for only 7 days (*females:* 48.1% vs. 49.4%; *males:* 52.3% vs. 50.4%). 14 days of CXCL1 neutralization decreased neutrophil infiltration (*females:* 0.43 vs. 5.21 cells/high power field (hpf), *p* = 0.04; *males:* 0.00 vs. 4.42 cells/hpf, *p* = 0.04) and increased reparative M2 macrophages (*females:* 2.25 vs. 0.79 cells/hpf, *p* = 0.03; *males:* 2.00 vs. 0.27 cells/hpf, *p* = 0.02).

**Conclusions:**

CXCL1 neutralization for 14 or 21 days improved aneurysm healing in female and male mice. 14 days of CXCL1 neutralization decreased neutrophil infiltration and increased M2 macrophage polarization. Systemic CXCL1 neutralization is a promising potential therapy to improve aneurysm healing by modulating the inflammatory response after coiling.

## Introduction

Intracranial aneurysm (IA) rupture results in subarachnoid hemorrhage, a life-threatening condition with significant morbidity and mortality. In well-selected patients, coiling remains a commonly used endovascular technique for treatment of unruptured IAs. One drawback, however, is that up to 26.3% of coiled aneurysms demonstrate recanalization which may require retreatment (Futchko et al., 2018; Teleb et al., 2014). Thus, it is imperative to develop durable techniques for complete aneurysm occlusion. Attempts at modifying aneurysm coils have had mixed results on recurrence rates (McDougall et al., 2014; Molyneux et al., 2012; Bendok et al., 2020). Another strategy can be augmenting aneurysm healing after coiling using a drug. Developing pharmacologic therapeutics, however, requires uncovering novel mechanisms involved in aneurysm healing.

Based on histopathological studies, the healing process after coil embolization is similar to wound healing (Bavinzski et al., 1999). After aneurysm coiling, the spaces between the coils are filled with thrombus (Bavinzski et al., 1999). In aneurysms that become complete occluded, this process is followed by inflammatory cell infiltration, connective tissue proliferation, and fibrous scar formation (Bavinzski et al., 1999; Brinjikji et al., 2015). Importantly, resolution of inflammatory processes is a key step in transitioning to formation of a stable scar (Brinjikji et al., 2015; Landen et al., 2016; Zhao et al., 2016). The inflammatory pathways specific to aneurysm healing are not fully understood. We have demonstrated that there is increased expression of the pro-inflammatory chemokine CXCL1, a potent neutrophil chemoattractant, in IAs (Nowicki et al., 2014). Persistent neutrophil activity and unresolved inflammation have been implicated in nonhealing wounds (Landen et al., 2016; Zhao et al., 2016; Soehnlein et al., 2017). Further, in our murine IA model, CXCL1 neutralization facilitated macrophage phenotype switching from “pro-inflammatory” M1 macrophages to “reparative” M2 macrophages (Nowicki et al., 2018). Indeed, M2 macrophages are associated with improved wound healing (Hesketh et al., 2017).

Given (1) the increased expression of CXCL1 in IAs and (2) the role of CXCL1 in modulating key leukocytes involved in wound healing, we hypothesized that CXCL1 neutralization would improve aneurysm healing after coil implantation. We also investigated changes in neutrophil infiltration and macrophage phenotypes to elucidate mechanisms of CXCL1-mediated aneurysm healing. The findings of this study are clinically relevant as developing novel pharmacotherapeutic options that target inflammatory mediators involved in aneurysm healing may serve as adjunctive therapy to endovascular coiling.

## Materials and methods

### Animals

Female and male C57BL/6 mice (8–12 weeks, Charles River, Massachusetts, USA) were used for all experiments. Animal studies were approved by the Institutional Animal Care and Use Committee and complied with both the Guide for the Care and Use of Laboratory Animals and the ARRIVE guidelines (National Research Council, 1996). Animals received food and water *ad libitum* and were housed in a room with 12-h light/dark cycling. A total of 75 female mice and 75 male mice were used across all experiments. Eight female mice and 10 male mice were excluded due to insufficient aneurysm formation.

### CXCL1 expression in murine carotid artery aneurysms

Mice were randomized to undergo either elastase surgery to create a saccular carotid artery aneurysm or sham surgery as previously described (Hoh et al., 2010). Briefly, mice were anesthetized using intraperitoneal injections of ketamine/xylazine. Through a midline neck incision, the right common carotid artery (RCCA) was isolated. The RCCA was bathed extravascularly in elastase (10 U/mL in phosphate-buffered saline (PBS); Worthington Biochemical Corporation, New Jersey, USA) for 20 min and was then occluded distally via cauterization. In the sham group, the RCCA was bathed in PBS and the distal end was not cauterized. Twenty one days later, mice were euthanized via cardiac perfusion of PBS followed by 4% paraformaldehyde (PFA) (Santa Cruz Biotechnology, Texas, USA). The RCCA was harvested, soaked in 4% PFA overnight, transferred to 18% sucrose overnight, and then frozen in Optimal Cutting Temperature (OCT) using dry ice and 2-methylbutane. A cryostat was then used to collect 5 μm sections. Immunohistochemistry was used to assess CXCL1 expression (see “Immunohistochemistry” section). Sections were imaged using a Leica DM2500 optical microscope (Leica Microsystems Inc., Illinois, USA); 40x images were used for quantification. The mean gray value was measured using ImageJ software and results were reported in relative fluorescence units (RFU) (Schneider et al., 2012).

### Murine carotid artery coil embolization model

We used our aneurysm coiling model to study CXCL1-mediated aneurysm healing. Saccular aneurysms were created in female and male mice as above. Twenty one days later, aneurysms were coiled using platinum coils coated in 50:50 poly (lactic-co-glycolic acid) (PLGA) (Sigma-Aldrich, Missouri, USA) (Schneider et al., 2012). Briefly, the RCCA aneurysm was isolated as in the initial aneurysm creation surgery. Blood flow through the aneurysm was occluded both proximally and distally using 3-0 sutures (Ethicon, New Jersey, USA). Then, an arteriotomy was made in the proximal RCCA, a PLGA coil was implanted, and the arteriotomy was closed using cauterization. Finally, the proximal and distal sutures were removed.

### CXCL1 neutralization on intrasaccular tissue ingrowth in coiled aneurysms

Once we confirmed increased CXCL1 expression in aneurysms compared to normal vasculature, we investigated the effect of systemic CXCL1 neutralization on tissue ingrowth after aneurysm coiling. Saccular aneurysms were created in female and male mice as above. Twenty one days later, aneurysms were coiled using platinum coils coated in 50:50 poly (lactic-co-glycolic acid) (PLGA) (Sigma-Aldrich, Missouri, USA) (Hoh et al., 2011). Mice were randomly assigned to receive blinded intraperitoneal injections of either CXCL1 neutralizing antibody (100 μg/mL, R&D Systems, Minnesota, USA) or isotype-matched IgG control (100 μg/mL, R&D Systems, Minnesota, USA). An intraperitoneal route was selected given its technical feasibility, high frequency of injections required for our study design, and well-established protocols for intraperitoneal injections that both we and others have used (Hourani et al., 2018). While this does subject the antibodies to first pass metabolism, we have previously demonstrated intraperitoneal injections to be feasible in our aneurysm model (Hourani et al., 2018). Injections were started 2 days prior to coiling and continued every 2 days, including the day of coiling, until the experimental endpoint. The rationale for initiating CXCL1 neutralization 2 days prior to aneurysm coiling was to ensure therapeutic effect by the time of aneurysm coiling surgeries. This dosing regimen was based on previous studies of systemic CXCL1 neutralization in mice (Nowicki et al., 2014, 2018). CXCL1 neutralization or IgG control was given for an additional 7, 14, or 21 days post-coiling. These mice were sacrificed 21 days after coiling and coiled aneurysms were harvested and sectioned as above.

### Intrasaccular tissue ingrowth measurements

To assess the efficacy of CXCL1 neutralization, intrasaccular tissue ingrowth was measured using histology. Three sections were chosen at random from each animal in mice treated for 7, 14, or 21 days and sacrificed at 21 days post-coiling. These sections were stained with hematoxylin and eosin and imaged. Tissue ingrowth was then measured using Image Pro (Media Cybernetics, Maryland, USA). To calculate the percentage of tissue ingrowth, the area of the lumen not filled by tissue ingrowth was subtracted from the total area inside the aneurysm wall. This calculation can be summarized as: $ingrowth\;\left(\%\right)\;=\;\frac{outer\;area-inner\;area}{outer\;area}x\;100$. The average of three distinct sections per animal was used for statistical analyses.

### Immunohistochemistry

CXCL1 expression and inflammatory cell infiltration of neutrophils, M1 macrophages, and M2 macrophages was assessed using immunohistochemistry. Tissue sections underwent heat-based antigen retrieval using DAKO (Agilent, California, USA) followed by blocking with 4% horse serum. Primary antibodies included: CXCL1 (ab86436, abcam, Massachusetts, USA), NIMP-R14 (ab2557, abcam, Massachusetts, USA), F4/80 (MCA497, Bio-Rad, California, USA), iNOS (ab15323, abcam, Massachusetts, USA), and arginase 1 (ab60176, abcam, Massachusetts, USA). Secondary antibodies include the following Alexa Fluor antibodies: donkey anti-rat 594, donkey anti-rabbit 594, donkey anti-goat 488, and donkey anti-rabbit 488 (ThermoFisher, Massachusetts, USA). Following secondary antibody incubation, slides were mounted using VECTASHIELD with 4^‘^,6-diamidino-2-phenylindole (DAPI) (Vector Laboratories, California, USA) as a nuclear counterstain. For inflammatory cell quantification, three non-overlapping regions were imaged from each animal at 40x. NIMP-R14 was used as a neutrophil marker and F4/80 was used to identify macrophages. M1 macrophages were identified by co-staining for F4/80 and iNOS and M2 macrophages were identified by co-staining for F4/80 and arginase 1. To quantify total macrophage infiltration, we recorded the total number of F4/80^+^ cells, including those without co-staining with iNOS or arginase 1, when counting M1 and M2 macrophages and used the average for statistical analyses. Positive controls were included with all staining experiments.

### Blinding, randomization and statistical analysis

Animals were randomly assigned to different experimental groups prior to initiating any surgeries. Ingrowth measurements and cell counts were performed by two blinded observers and all image files were deidentified and randomized. The Mann-Whitney U test used to detect differences in CXCL1 expression, tissue ingrowth, and inflammatory cell counts. One-way ANOVA with Newman-Keuls *post hoc* test was used to compare tissue ingrowth between different lengths of treatment within an experimental group. Analyses were performed using GraphPad Prism and results are reported with standard error of the mean. The threshold for statistical significance was set to *p* < 0.05.

## Results

### CXCL1 expression is increased in murine carotid artery aneurysms

**Figure 1:**
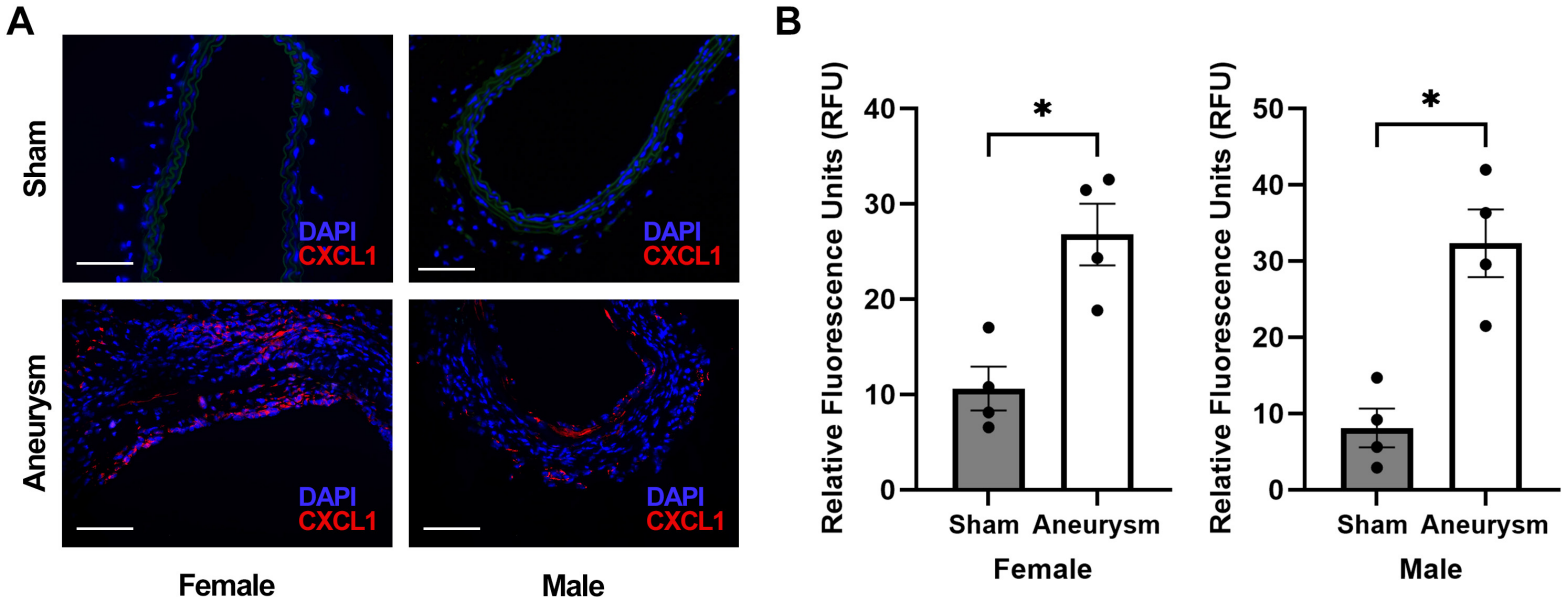
CXCL1 expression in murine carotid artery aneurysm model. **(A)** Representative immunohistochemistry images at 40x (scale bar = 50 μm) of female sham-operated carotid arteries, female carotid aneurysms, male sham-operated carotid arteries, and male carotid aneurysms. DAPI (blue, nuclear staining); CXCL1 (red); Autofluorescence (green). **(B)** CXCL1 expression is increased in both females and males with aneurysms compared to sham-operated carotid arteries (*females:* 26.8 ± 3.22 vs. 10.6 ± 2.31 RFU, *n* = 4 each, *p* = 0.03; *males:* 32.3 ± 4.42 vs. 8.1 ± 2.55 RFU, *n* = 4 each, *p* = 0.03). There is no difference in aneurysm CXCL1 expression between females and males (26.8 ± 3.22 vs. 32.3 ± 4.42 RFU, *n* = 4 each, *p* = 0.49). *p < 0.05.

Using immunohistochemistry, we first confirmed that both female and male mice had increased CXCL1 expression in carotid artery aneurysms compared to the normal carotid arteries in sham-operated mice (females: 26.8 ± 3.22 vs. 10.6 ± 2.31 RFU, *n* = 4 each, *p* = 0.03; males: 32.3 ± 4.42 vs. 8.1 ± 2.55 RFU, *n* = 4 each, *p* = 0.03) (Figures 1A, B). There was no difference in CXCL1 expression between females and males with aneurysms (26.8 ± 3.22 vs. 32.3 ± 4.42 RFU, *n* = 4 each, *p* = 0.49) (Figure 1B).

### CXCL1 neutralization for 14 and 21 days increases tissue ingrowth in coiled aneurysms

**Figure 2:**
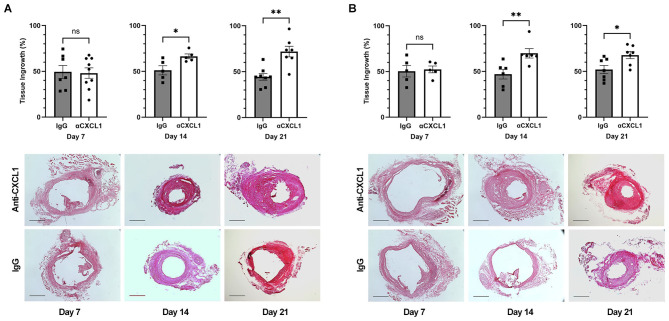
Tissue ingrowth with varying lengths of treatment with CXCL1 neutralization or IgG control post-coiling. **(A)** In females, CXCL1 neutralization increases tissue ingrowth compared to IgG when given for 14 days (66.4% ± 2.60 vs. 51.2% ± 5.01; *n* = 5 each; *p* = 0.03) and 21 days (71.9% ± 5.79 vs. 44.3% ± 5.10; *n* = 7 and 8; *p* = 0.002) but not when given for 7 days (48.1% ± 5.71 vs. 49.4% ± 7.03; *n* = 9 and 7; *p* = 0.92). Representative images of tissue growth with 7, 14, and 21 days of CXCL1 neutralization or IgG control are below. Scale bar = 200 μm. **(B)** In males, CXCL1 neutralization increased tissue ingrowth compared to IgG when given for 14 days (69.8% ± 5.13 vs. 47.0% ± 5.29; *n* = 6 each; *p* = 0.004) and 21 days (67.8% ± 3.93 vs. 53.8% ± 4.25; *n* = 7 and 8; *p* = 0.002) but not when given for 7 days (52.3% ± 3.68 vs. 50.4% ± 6.33; *n* = 5 each; *p* = 1.00). Representative images of tissue growth with 7, 14, and 21 days of CXCL1 neutralization or IgG control are below. Scale bar = 200 μm. *p < 0.05, **p < 0.01.

In female mice, CXCL1 neutralization increases tissue ingrowth compared to IgG when given for 14 days (66.4% ± 2.60 vs. 51.2% ± 5.01; *n* = 5 each; *p* = 0.03) and 21 days (71.9% ± 5.79 vs. 44.3% ± 5.10; *n* = 7 and 8; *p* = 0.002) but not when given for 7 days (48.1% ± 5.71 vs. 49.4% ± 7.03; *n* = 9 and 7; *p* = 0.92) (Figures 2A, B). In male mice, CXCL1 neutralization increased tissue ingrowth compared to IgG when given for 14 days (69.8% ± 5.13 vs. 47.0% ± 5.29; *n* = 6 each; *p* = 0.004) and 21 days (67.8% ± 3.93 vs. 53.8% ± 4.25; *n* = 7 and 8; *p* = 0.02) but not when given for 7 days (52.3% ± 3.68 vs. 50.4% ± 6.33; *n* = 5 each; *p* = 1.00) (Figures 2A, B).

### Effect of CXCL1 neutralization on neutrophil infiltration

**Figure 3:**
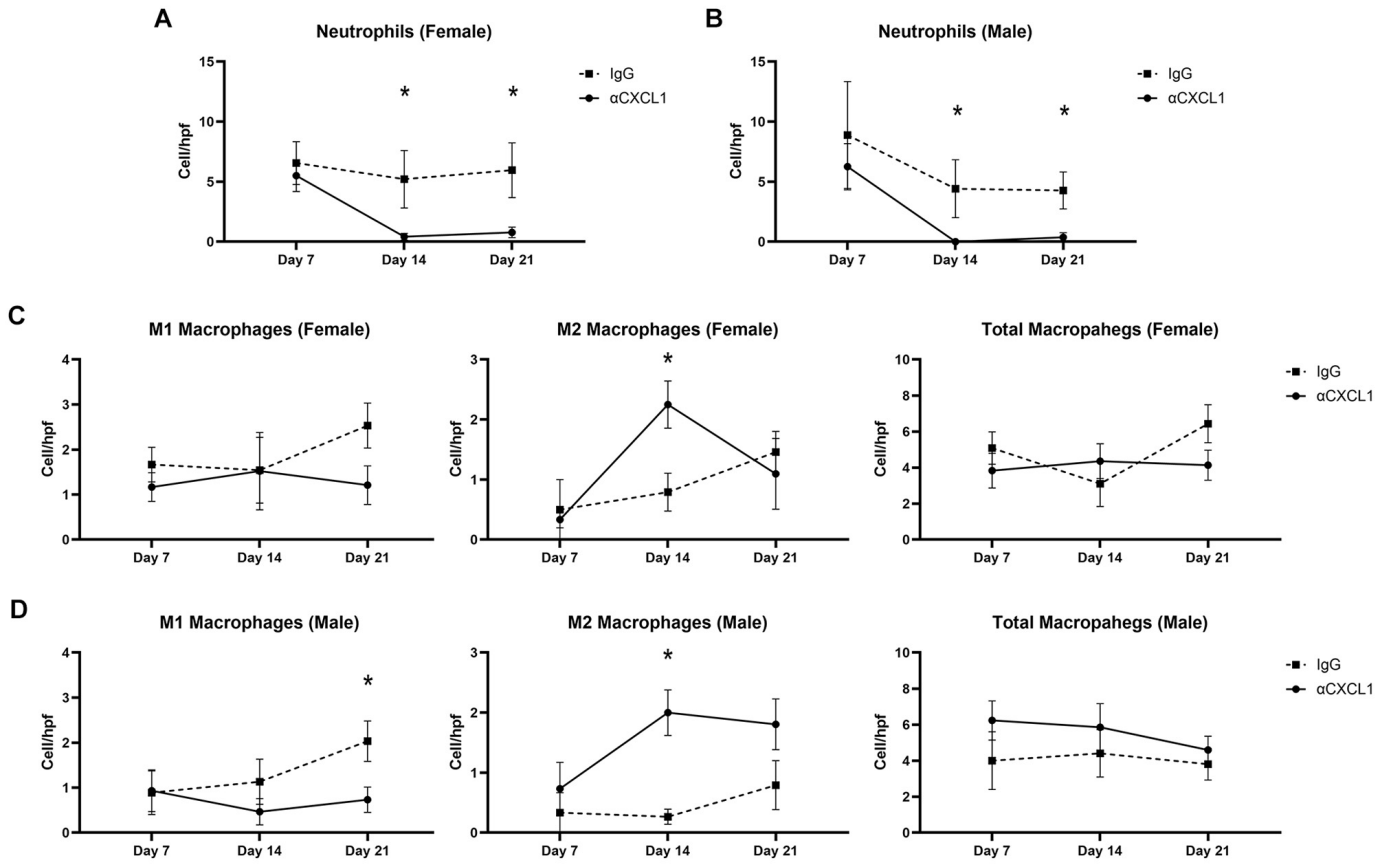
Analysis of neutrophil filtration and macrophage phenotype after coil implantation. **(A)** In females, CXCL1 neutralization significantly decreased neutrophil infiltration compared to IgG control at 14 days post-coiling (0.43 ± 0.28 vs. 5.21 ± 2.40 cells/hpf, *n* = 7 and 8, *p* = 0.04) and at 21 days post-coiling (0.79 ± 0.43 vs. 5.96 ± 2.28 cells/hpf; *n* = 7 and 4, *p* = 0.03). CXCL1 neutralization did not affect neutrophil chemotaxis compared to IgG at 7 days post-coiling (5.50 ± 1.31 vs. 6.56 ± 1.79 cells/hpf, *n* = 4 and 3, *p* = 0.72). **(B)** In males, there were significantly less neutrophils with CXCL1 neutralization compared to IgG at 14 days post-coiling (0.00 ± 0.00 vs. 4.42 ± 2.41 cells/hpf, *n* = 5 and 4, *p* = 0.04) and 21 days post-coiling (0.38 ± 0.38 vs. 4.27 ± 1.54 cells/hpf, *n* = 7 and 8, *p* = 0.03) but not at 7 days (6.25 ± 1.92 vs. 8.89 ± 4.45 cells/hpf, *n* = 4 and 3, *p* = 0.63). **(C)** In females, CXCL1 neutralization did not affect M1 macrophage polarization compared to IgG at 7 days post-coiling (1.17 ± 0.32 vs. 1.67 ± 0.38 cells/hpf, *n* = 4 and 3, *p* = 0.37), 14 days after coiling (1.52 ± 0.86 vs. 1.54 ± 0.73 cells/hpf, *n* = 4 and 4, *p* = 1.00), and 21 days after coiling (1.21 ± 0.43 vs. 2.53 ± 0.50 cells/hpf, *n* = 7 and 4, *p* = 0.22). CXCL1 neutralization significantly increased the number of M2 macrophages 14 days post-coiling (2.25 ± 0.39 vs. 0.79 ± 0.31 cells/hpf, *n* = 4 each, *p* = 0.03) but not at 7 days after coiling (0.33 ± 0.14 vs. 0.50 ± 0.50 cells/hpf, *n* = 4 and 3, *p* = 0.85) or at 21 days post-coiling (1.10 ± 0.59 vs. 1.46 ± 0.34 cells/hpf, *n* = 7 and 4, *p* = 0.34). Total macrophage burden did not differ between CXCL1 neutralization and IgG control groups 7 days after coiling (3.83 ± 0.97 vs. 5.08 ± 0.90 cells/hpf, *n* = 4 and 3, *p* = 0.24), 14 days after coiling (4.35 ± 0.96 vs. 3.10 ± 1.26 cells/hpf, *n* = 4 each, *p* = 0.32), or 21 days after coiling (4.02 ± 0.82 vs. 6.43 ± 1.06 cells/hpf, *n* = 7 and 4, *p* = 0.12). **(D)** In males, CXCL1 neutralization did not affect M1 macrophage polarization 7 days after coiling (0.93 ± 0.46 vs. 0.89 ± 0.48 cells/hpf, *n* = 5 and 3, *p* = 1.00) or at 14 days (0.47 ± 0.29 vs. 1.13 ± 0.50 cells/hpf, *n* = 5 each, *p* = 0.44). At 21 days, however, mice treated with CXCL1 neutralization had significantly fewer M1 macrophages (0.73 ± 0.28 vs. 2.03 ± 0.45 cells/hpf, *n* = 5 each, *p* = 0.045). CXCL1 neutralization increased M2 macrophage polarization at 14 days post-coiling (2.00 ± 0.38 vs. 0.27 ± 0.12 cells/hpf, *n* = 5 each, *p* = 0.02) but had no effect at 7 days (0.73 ± 0.44 vs. 0.33 ± 0.33 cells/hpf, *n* = 5 and 3, *p* = 0.63) or at 21 days (1.81 ± 0.42 vs. 0.79 ± 0.41 cells/hpf, *n* = 6 and 8, *p* = 0.13). CXCL1 neutralization had no effect on total macrophage burden at 7 days (6.23 ± 1.09 vs. 4.00 ± 1.60 cells/hpf, *n* = 5 and 3, *p* = 0.19), 14 days (5.85 ± 1.31 vs. 4.41 ± 1.31 cells/hpf, *n* = 5 each, *p* = 0.38), or at 21 days (4.59 ± 0.77 vs. 3.81 ± 0.88 cells/hpf, *n* = 5 each, *p* = 0.52). *p < 0.05.

There was a significant reduction in neutrophil infiltration at 14 days post-coiling with CXCL1 neutralization compared to IgG in females (0.43 ± 0.28 vs. 5.21 ± 2.40 cells/hpf, *n* = 7 and 8, *p* = 0.04) and males (0.00 ± 0.00 vs. 4.42 ± 2.41 cells/hpf, *n* = 5 and 4, *p* = 0.04) (Figures 3A, B). Similarly, with 21 days of CXCL1 neutralization, both female (0.79 ± 0.43 vs. 5.96 ± 2.28 cells/hpf; *n* = 7 and 4, *p* = 0.03) and male (0.38 ± 0.38 vs. 4.27 ± 1.54 cells/hpf, *n* = 7 and 8, *p* = 0.03) aneurysms had significantly fewer neutrophils compared to control (Figures 3A, B). At 7 days post-coiling, however, CXCL1 neutralization did not affect neutrophil filtration in females (5.50 ± 1.31 vs. 6.56 ± 1.79 cells/hpf, *n* = 4 and 3, *p* = 0.72) or males (6.25 ± 1.92 vs. 8.89 ± 4.45 cells/hpf, *n* = 4 and 3, *p* = 0.63) (Figures 3A, B).

### Effect of CXCL1 neutralization on macrophage polarization and infiltration

In both females and males, there was no difference in the number of M1 macrophages between CXCL1 neutralization and IgG control 7 days after coiling (female; 1.17 ± 0.32 vs. 1.67 ± 0.38 cells/hpf, *n* = 4 and 3, *p* = 0.37: male; 0.93 ± 0.46 vs. 0.89 ± 0.48 cells/hpf, *n* = 5 and 3, *p* = 1.00) and 14 days after coiling (female; 1.52 ± 0.86 vs. 1.54 ± 0.73 cells/hpf, *n* = 4 and 4, *p* = 1.00: male; 0.47 ± 0.29 vs. 1.13 ± 0.50 cells/hpf, *n* = 5 each, *p* = 0.44) (Figures 3C, D). At 21 days post-coiling, CXCL1 neutralization significantly reduced M1 macrophages in males (0.73 ± 0.28 vs. 2.03 ± 0.45 cells/hpf, *n* = 5 each, *p* = 0.045), but not females (1.21 ± 0.43 vs. 2.53 ± 0.50 cells/hpf, *n* = 7 and 4, *p* = 0.22) (Figures 3C, D).

At 14 days post-coiling, female (2.25 ± 0.39 vs. 0.79 ± 0.31 cells/hpf, *n* = 4 each, *p* = 0.03) and male (2.00 ± 0.38 vs. 0.27 ± 0.12 cells/hpf, *n* = 5 each, *p* = 0.02) mice treated with CXCL1 neutralization had more M2 macrophages compared to IgG control-treated mice (Figures 3C, D). CXCL1 neutralization did not significantly affect the number of M2 macrophages at 7 days (female; 0.33 ± 0.14 vs. 0.50 ± 0.50 cells/hpf, *n* = 4 and 3, *p* = 0.85: male; 0.73 ± 0.44 vs. 0.33 ± 0.33 cells/hpf, *n* = 5 and 3, *p* = 0.63) or 21 days post-coiling (female; 1.10 ± 0.59 vs. 1.46 ± 0.34 cells/hpf, *n* = 7 and 4, *p* = 0.34: male; 1.81 ± 0.42 vs. 0.79 ± 0.41 cells/hpf, *n* = 6 and 8, *p* = 0.13) ( fig3Figures 3C, D).

There was no significant change in total macrophage infiltration between CXCL1 neutralization and IgG control at 7 (female; 3.83 ± 0.97 vs. 5.08 ± 0.90 cells/hpf, *n* = 4 and 3, *p* = 0.24: male 6.23 ± 1.09 vs. 4.00 ± 1.60 cells/hpf, *n* = 5 and 3, *p* = 0.19), 14 (female; 4.35 ± 0.96 vs. 3.10 ± 1.26 cells/hpf, *n* = 4 each, *p* = 0.32: male; 5.85 ± 1.31 vs. 4.41 ± 1.31 cells/hpf, *n* = 5 each, *p* = 0.38), or 21 days (female; 4.02 ± 0.82 vs. 6.43 ± 1.06 cells/hpf, *n* = 7 and 4, *p* = 0.12: male; 4.59 ± 0.77 vs. 3.81 ± 0.88 cells/hpf, *n* = 5 each, *p* = 0.52) post-coiling (Figures 3C, D).

## Discussion

Aneurysm recurrence is a significant drawback of coiling and novel strategies toward a durable treatment option are needed. One such strategy could include augmenting the healing process after coiling using a drug. Development of such strategies, however, requires a better understanding of the mechanisms underlying aneurysm healing after coiling. Studies using both human autopsy specimens and animal models support the critical role of inflammation after coil embolization and suggest that intraluminal healing may share similarities with wound healing (Bavinzski et al., 1999; Brinjikji et al., 2015; Hoh et al., 2011, 2018; Hosaka et al., 2017; Hourani et al., 2018). Neutrophils are the first responders to inflammation or injury sites, recruited through chemotactic factors (Bavinzski et al., 1999; Mayadas et al., 2014). We previously identified the critical role of neutrophils in intracranial aneurysm pathophysiology (Patel et al., 2023). As they aggregate and become activated via proinflammatory signals, they recruit macrophages based on the stimulus provided (Bavinzski et al., 1999; Brinjikji et al., 2015; Wajima et al., 2020).

We have previously shown that CXCL1, an inflammatory cytokine, is highly expressed in human aneurysms and plays a role in both neutrophil infiltration and macrophage polarization (Nowicki et al., 2014, 2018). In this study, we confirmed increased expression of CXCL1 in both female and male murine carotid artery aneurysms. This finding became the impetus for studying the role of CXCL1 in aneurysm healing after coiling. Using our previously established model, we implanted PLGA-coated coils into carotid artery aneurysms in female and male mice. These coils served as a scaffold on which intraluminal tissue ingrowth can occur. Mice received systemic CXCL1 neutralizing antibody or IgG control via intraperitoneal injections for either 7, 14, or 21 days post-coiling. CXCL1 neutralization for 14 days and 21 days improved the amount of tissue ingrowth compared to IgG control in both females and males. Next, we sought to understand the mechanism underlying the efficacy of CXCL1 neutralization in promoting intraluminal tissue ingrowth when administered for 14 or 21 days, but not 7 days. To address this gap in knowledge, we analyzed changes in key inflammatory cell populations at 7, 14, and 21 days post-coiling between mice treated with CXCL1 neutralization and those treated with IgG control. CXCL1 is a potent neutrophil chemoattractant and neutrophils may be detrimental to tissue healing processes through collateral tissue damage (Zhao et al., 2016; Soehnlein et al., 2017). Indeed, CXCL1 neutralization decreased neutrophil infiltration at 14 and 21 days post-coiling in females and males.

In our murine intracranial aneurysm model, we demonstrated previously that CXCL1 affects macrophage phenotype switching between “pro-inflammatory” M1 macrophages and “reparative” M2 macrophages (Nowicki et al., 2018). In chronic nonhealing wounds, macrophages are predominantly M1 macrophages (Hesketh et al., 2017). Conversely, M2 macrophages contribute to tissue repair and scar formation (Hesketh et al., 2017). Scar formation is a desirable outcome in coiled aneurysms to prevent recurrence. In this study, we found that female and male mice treated with CXCL1 neutralization had increased M2 macrophages at 14 days post-coiling but not at 7 or 21 days post-coiling. CXCL1 neutralization decreased M1 macrophages only at 21 days post-coiling in males. These findings suggest that an increase in “reparative” M2 macrophages during the intermediate stage of aneurysm healing may be responsible for the increased tissue ingrowth seen with 14 days of CXCL1 neutralization. Notably, total macrophage infiltration was not affected by CXCL1 neutralization.

In summary, CXCL1 neutralization for 14 and 21 days improves aneurysm healing in both females and males. Correspondingly, CXCL1 neutralization decreases neutrophil infiltration and shifts macrophage phenotypes toward “reparative” M2 macrophages at 14 days post-coiling. This correlation suggests the mechanism underlying the efficacy of CXCL1 neutralization is through regulation of neutrophil chemotaxis and macrophage polarization at 14 days post-coiling. Thus, CXCL1 is a novel therapeutic target to improve intraluminal aneurysm healing. Given the translational applicability of our coiling model, targeting this CXCL1 pathway is a feasible option to improve outcomes after coiling and prevent aneurysm recurrence. The findings of this study have direct clinical applications in that CXCL1 can be inhibited, such as with a pill, in patients undergoing coiling to improve the chances their aneurysm will be cured. Further, our results may have broad clinical applicability given that we accounted for sex differences by using both female and male mice.

### Limitations and future directions

This study had some limitations. First, our coiling model uses an extracranial vessel. Nonetheless, the purpose of this model is to study intraluminal tissue healing. Next, our CXCL1 blockade vs. IgG control dosing regimen started 2 days prior to aneurysm coiling. Our results demonstrate that neutrophil cells remain elevated and M2 macrophages remain scarce until 14 days of CXCL1 blockade. From a clinical translational perspective, future studies are required to determine if persistent CXCL1 blockade is needed from the time of aneurysm treatment or if treatment can be started during latter parts of the inflammatory cascade (e.g., 3–5 days after aneurysm treatment).

Further, while we investigated the effect of CXCL1 neutralization on neutrophil infiltration and macrophage phenotypes, additional studies are needed to directly study the relative contributions of each. Future studies in which neutrophils and M2 macrophages are directly depleted would provide more definitive proof of causality. In addition, we have previously investigated the effects of local modulation of inflammatory pathways (Hoh et al., 2011, 2018). Future studies targeting local CXCL1 blockade through drug-eluting coils will be of significant translational value. Additionally, there is growing evidence that IA pathophysiology is unique between females and males. While the intent of this study was not direct comparison between females and males, future studies in this area would be of great clinical significance.

## Disclosure

This work was supported by the following sources of support awarded to BLH: NIH R01-NS083673, R01-NS110710, R01-NS124620, the Brain Aneurysm Foundation, the James and Brigitte Marino Family Professorship Endowment, the Eblen Research Endowment, the Christine Desmond Fund, and the St. George Family Fund. The authors of this study have no relevant financial disclosures or conflicts of interest.

## Data Availability

The original contributions presented in the study are included in the article/supplementary material, further inquiries can be directed to the corresponding author.

## References

[B1] Bavinzski G. Talazoglu V. Killer M. Richling B. Gruber A. Gross C. E. . (1999). Gross and microscopic histopathological findings in aneurysms of the human brain treated with Guglielmi detachable coils. J. Neurosurg. 91, 284–293. doi: 10.3171/jns.1999.91.2.0284 10433317

[B2] Bendok B. R. Abi-Aad K. R. Ward J. D. Kniss J. F. Kwasny M. J. Rahme R. J. . (2020). The Hydrogel Endovascular Aneurysm Treatment Trial (HEAT): a randomized controlled trial of the second-generation hydrogel coil. Neurosurgery 86, 615–624. doi: 10.1093/neuros/nyaa006 32078692 PMC7534546

[B3] Brinjikji W. Kallmes D. F. Kadirvel R. (2015). Mechanisms of healing in coiled intracranial aneurysms: a review of the literature. AJNR Am. J. Neuroradiol. 36, 1216–1222. doi: 10.3174/ajnr.A4175 25430855 PMC4939243

[B4] Futchko J. Starr J. Lau D. Leach M. R. Roark C. Pandey A. S. . (2018). Influence of smoking on aneurysm recurrence after endovascular treatment of cerebrovascular aneurysms. J. Neurosurg. 128, 992–998. doi: 10.3171/2016.12.JNS161625 28644100

[B5] Hesketh M. Sahin K. B. West Z. E. Murray R. Z. (2017). Macrophage phenotypes regulate scar formation and chronic wound healing. Int. J. Mol. Sci. 18:1545. doi: 10.3390/ijms18071545 28714933 PMC5536033

[B6] Hoh B. L. Fazal H. Z. Hourani S. Li M. Lin L. Hosaka K. (2018). Temporal cascade of inflammatory cytokines and cell-type populations in monocyte chemotactic protein-1 (MCP-1)-mediated aneurysm healing. J. Neurointerv. Surg. 10, 301–305. doi: 10.1136/neurintsurg-2017-013063 28450456 PMC6194857

[B7] Hoh B. L. Hosaka K. Downes D. P. Nowicki K. W. Fernandez C. E. Batich C. D. . (2011). Monocyte chemotactic protein-1 promotes inflammatory vascular repair of murine carotid aneurysms via a macrophage inflammatory protein-1alpha and macrophage inflammatory protein-2-dependent pathway. Circulation 124, 2243–2252. doi: 10.1161/CIRCULATIONAHA.111.036061 22007074 PMC3217188

[B8] Hoh B. L. Velat G. J. Wilmer E. N. Hosaka K. Fisher R. C. Scott E. W. (2010). A novel murine elastase saccular aneurysm model for studying bone marrow progenitor-derived cell-mediated processes in aneurysm formation. Neurosurgery 66, 544–550. doi: 10.1227/01.NEU.0000365616.46414.2B 20173550 PMC2905737

[B9] Hosaka K. Rojas K. Fazal H. Z. Schneider M. B. Shores J. Federico V. . (2017). Monocyte chemotactic protein-1-interleukin-6-osteopontin pathway of intra-aneurysmal tissue healing. Stroke 48, 1052–1060. doi: 10.1161/STROKEAHA.116.015590 28292871 PMC5390817

[B10] Hourani S. Motwani K. Wajima D. Fazal H. Jones C. H. Doré S. . (2018). Local delivery is critical for monocyte chemotactic protein-1 mediated site-specific murine aneurysm healing. Front. Neurol. 9:158. doi: 10.3389/fneur.2018.00158 29615957 PMC5868072

[B11] Landen N. X. Li D. Stahle M. (2016). Transition from inflammation to proliferation: a critical step during wound healing. Cell. Mol. Life Sci. 73, 3861–3885. doi: 10.1007/s00018-016-2268-0 27180275 PMC5021733

[B12] Mayadas T. N. Cullere X. Lowell C. A. (2014). The multifaceted functions of neutrophils. Annu. Rev. Pathol. 9, 181–218. doi: 10.1146/annurev-pathol-020712-164023 24050624 PMC4277181

[B13] McDougall C. G. Johnston S. C. Gholkar A. Barnwell S. L. Vazquez Suarez J. C. Massó Romero J. . (2014). Bioactive versus bare platinum coils in the treatment of intracranial aneurysms: the MAPS (Matrix and Platinum Science) trial. AJNR Am. J. Neuroradiol. 35, 935–942. doi: 10.3174/ajnr.A3857 24481333 PMC7964535

[B14] Molyneux A. J. Clarke A. Sneade M. Mehta Z. Coley S. Roy D. . (2012). Cerecyte coil trial: angiographic outcomes of a prospective randomized trial comparing endovascular coiling of cerebral aneurysms with either cerecyte or bare platinum coils. Stroke 43, 2544–2550. doi: 10.1161/STROKEAHA.112.657254 22836352

[B15] National Research Council (1996). Guide for the Care and Use of Laboratory Animals. Washington, DC: The National Academies Press.

[B16] Nowicki K. W. Hosaka K. He Y. McFetridge P. S. Scott E. W. Hoh B. L. (2014). Novel high-throughput in vitro model for identifying hemodynamic-induced inflammatory mediators of cerebral aneurysm formation. Hypertension 64, 1306–1313. doi: 10.1161/HYPERTENSIONAHA.114.03775 25225207 PMC4231007

[B17] Nowicki K. W. Hosaka K. Walch F. J. Scott E. W. Hoh B. L. (2018). M1 macrophages are required for murine cerebral aneurysm formation. J. Neurointerv. Surg. 10, 93–97. doi: 10.1136/neurintsurg-2016-012911 28196918 PMC7814362

[B18] Patel D. Dodd W. S. Lucke-Wold B. Chowdhury M. A. B. Hosaka K. Hoh B. L. (2023). Neutrophils: novel contributors to estrogen-dependent intracranial aneurysm rupture via neutrophil extracellular traps. J. Am. Heart Assoc. 12:e029917. doi: 10.1161/JAHA.123.029917 37889179 PMC10727420

[B19] Schneider C. A. Rasband W. S. Eliceiri K. W. (2012). NIH Image to ImageJ: 25 years of image analysis. Nat. Methods 9, 671–675. doi: 10.1038/nmeth.2089 22930834 PMC5554542

[B20] Soehnlein O. Steffens S. Hidalgo A. Weber C. (2017). Neutrophils as protagonists and targets in chronic inflammation. Nat. Rev. Immunol. 17, 248–261. doi: 10.1038/nri.2017.10 28287106

[B21] Teleb M. S. Pandya D. J. Castonguay A. C. Eckardt G. Sweis R. Lazzaro M. A. . (2014). Safety and predictors of aneurysm retreatment for remnant intracranial aneurysm after initial endovascular embolization. J. Neurointerv. Surg. 6, 490–494. doi: 10.1136/neurintsurg-2013-010836 23956245

[B22] Wajima D. Hourani S. Dodd W. Patel D. Jones C. Motwani K. . (2020). Interleukin-6 promotes murine estrogen deficiency-associated cerebral aneurysm rupture. Neurosurgery 86, 583–592. doi: 10.1093/neuros/nyz220 31264696 PMC7317988

[B23] Zhao R. Liang H. Clarke E. Jackson C. Xue M. (2016). Inflammation in chronic wounds. Int. J. Mol. Sci. 17:2085. doi: 10.3390/ijms17122085 27973441 PMC5187885

